# AI-powered neuroimaging markers: a new era in paediatric Leigh syndrome diagnosis

**DOI:** 10.1097/MS9.0000000000003999

**Published:** 2025-09-30

**Authors:** Raiha Yasser, Faiza Imran, Maliha Khalid, Muhammad Talha, Aminath Waafira

**Affiliations:** a Department of Medicine, Fatima Jinnah Medical University, lahore; b Department of Medicine, Fatima Memorial Hospital College of Medicine and Dentistry, Lahore; c Department of Medicine, Jinnah Sindh Medical University, karachi, Pakistan; d king Edward Medical University, lahore, Pakistan; e Department of Medicine, School of Medicine, Maldives National University, Male, Maldives

**Keywords:** artificial intelligence, Leigh syndrome, neuroimaging, pediatric neurology

## Abstract

Leigh syndrome is a severe pediatric mitochondrial disorder characterized by progressive neurological decline and epilepsy as a frequent manifestation. Traditional diagnostic approaches, including EEG and MRI, are often limited in their sensitivity and specificity, leading to diagnostic delays. Artificial intelligence (AI)-powered neuroimaging markers have emerged as promising tools that integrate multimodal patient data – including clinical history, imaging, and genetic sequencing – to enhance diagnostic precision and facilitate early intervention. Recent studies have demonstrated AI’s ability to classify epilepsy subtypes, automate EEG interpretation, and detect early neurodegenerative changes, underscoring its clinical potential. However, challenges such as algorithm transparency, dataset bias, and ethical concerns remain. Integrating AI-driven neurotechnology with existing modalities may significantly improve the early diagnosis and prognosis of Leigh syndrome, offering a pathway toward precision neurodiagnostics in pediatric care.

*To the Editor,*

Leigh syndrome (or subacute necrotizing encephalomyelopathy) is a severe neurodegenerative disorder that is characterized by symmetrical lesions extending from the basal ganglia and thalamus through the brainstem to the posterior columns of the spinal cord. It is a syndrome of mitochondrial disease that typically presents in pediatric patients, with mutations in mtDNA or nDNA. Epilepsy has been characterized as a typical phenotypic feature of mitochondrial diseases^[1]^.

Artificial intelligence (AI)-driven neurotechnology markers have shown promise in the prompt diagnosis of pediatric neurological disorders in clinical settings with enhances precision and accuracy. This phenomenon is due to the system’s ability of swift access and processing of patients’ data, including patient histories, neurological imaging results, and genetic sequencing reports. Expedited detection of debilitating neurological disorders can result in early intervention and improve patient care by developing new protocols to assist clinicians with more accuracy and efficiency, mitigating the disease progression and development of complications^[2,3]^.

A study by Soriano *et al* used machine learning algorithms to analyze and classify brain signals in generalized and focal epilepsy. They found that the system could differentiate between idiopathic generalized and focal epilepsy types with a high accuracy of 86%^[4]^. AI-based systems can automate the procedure of analysis of EEG and thereby result in a quick and accurate diagnosis^[5]^. Another study demonstrates that AI algorithms can be used to detect early signs of Alzheimer’s disease by studying the structural and functional MRI scans even before the clinical symptoms start to appear^[6]^.

Despite the fact that AI has shown promise in diagnosis, effective monitoring, and treatment of different neurological conditions and their phenotypes, there are still ethical, legal, and socioeconomic challenges ingrained in the pursuit of AI-driven healthcare^[7]^. One major challenge is the “black box” nature of many AI algorithms, especially those based on deep learning models. The inner workings are often opaque to clinicians, leading to concerns about the interpretability of AI-assisted diagnosis and treatments. This can lead to a lack of trust and adoption in clinical settings^[8]^. Moreover, an AI system is heavily dependent on the data it is trained on. So larger studies are required to correctly estimate the accuracy of the studies. As most of these studies were conducted on the Western population, this also raises concerns about the applicability of these methods to other populations.

In conclusion, Leigh syndrome has complex and variable clinical features, with epilepsy being a potent phenotypic manifestation. Traditional diagnostic tools like EEG and emerging modalities like fNIRS and NIRS emphasize the need for more targeted neurodiagnostic methods. The integration of AI-driven analysis with these neuroimaging techniques could enhance the sensitivity and specificity of early epilepsy detection in pediatric Leigh syndrome patients. AI can only augment the diagnostic precision and comprehensive insights, but not supplant. The exigency for efficient AI-driven neurotechnology markers for effective diagnosis and good prognosis in Leigh syndrome is imminent now. This letter to editor is in compliance with the TITAN guideline^[9]^.

## Data Availability

No data were generated for this manuscript.

## Research registration unique identifying number (UIN)

Name of the registry: N/A. Unique identifying number or registration ID: N/A. Hyperlink to your specific registration (must be publicly accessible and will be checked): N/A.
